# Sound localization in noisy contexts: performance, metacognitive evaluations and head movements

**DOI:** 10.1186/s41235-023-00530-w

**Published:** 2024-01-08

**Authors:** Chiara Valzolgher, Sara Capra, Elena Gessa, Tommaso Rosi, Elena Giovanelli, Francesco Pavani

**Affiliations:** 1 Center for Mind/Brain Sciences (CIMeC), University of Trento, Corso Bettini 31, 38068 Rovereto, TN Italy; 2 Department of Psychology and Cognitive Sciences (DiPSCo), University of Trento, Trento, Italy; 3 Centro Interuniversitario di Ricerca “Cognizione, Linguaggio e Sordità” (CIRCLeS), Trento, Italy; 4 Department of Physics, University of Trento, Trento, Italy

**Keywords:** Spontaneous behavior, Sound localization, Metacognitive evaluations, Effort, Confidence, Self-efficacy

## Abstract

Localizing sounds in noisy environments can be challenging. Here, we reproduce real-life soundscapes to investigate the effects of environmental noise on sound localization experience. We evaluated participants' performance and metacognitive assessments, including measures of sound localization effort and confidence, while also tracking their spontaneous head movements. Normal-hearing participants (N = 30) were engaged in a speech-localization task conducted in three common soundscapes that progressively increased in complexity: nature, traffic, and a cocktail party setting. To control visual information and measure behaviors, we used visual virtual reality technology. The results revealed that the complexity of the soundscape had an impact on both performance errors and metacognitive evaluations. Participants reported increased effort and reduced confidence for sound localization in more complex noise environments. On the contrary, the level of soundscape complexity did not influence the use of spontaneous exploratory head-related behaviors. We also observed that, irrespective of the noisy condition, participants who implemented a higher number of head rotations and explored a wider extent of space by rotating their heads made lower localization errors. Interestingly, we found preliminary evidence that an increase in spontaneous head movements, specifically the extent of head rotation, leads to a decrease in perceived effort and an increase in confidence at the single-trial level. These findings expand previous observations regarding sound localization in noisy environments by broadening the perspective to also include metacognitive evaluations, exploratory behaviors and their interactions.

**Supplementary Information:**

The online version contains supplementary material available at 10.1186/s41235-023-00530-w.

## Introduction

In everyday situations, we often find ourselves in noisy acoustic environments, such as busy city streets or crowded restaurants. These are common soundscapes (Grinfeder et al., 2022) in which auditory interference can result from single (e.g., Lorenzi et al., 1999 or Kopčo et al., 2010) or multiple sound sources (e.g., Brungart et al., 2005). While there is a wealth of research on the interfering effects of noise when listening to speech (e.g., Cherry, 1953; Shinn-Cunningham, 2008; Sumby & Pollack, 1954), less attention has been given to the interfering effects of noise on the ability to identify the positions of sound sources.

Previous studies on sound localization in noise have documented that with a single source of noise, localization accuracy degrades as the signal-to-noise ratio decreases. While this effect involves all dimensions of space (azimuth, elevation, and distance), noise affects mostly elevation, distance and front/back discrimination. This suggests that noise impacts primarily on the analysis of monaural spectral cues, which are essential for sound localization in these dimensions. For instance, Good and Gilkey (1996) tested participants in a sound localization task in quiet and in the presence of broadband noise at different signal-to-noise ratios (SNR). Target sounds were emitted by one of 239 possible loudspeakers, distributed all around the participants, while the noise was always located in front of them. They found that the presence of noise influenced participants’ ability to indicate the perceived direction of the signal mostly on the front/back dimension. Previous studies also showed that noise interference depends on source location relative to the signal (Lorenzi et al., 1999; Kopčo et al., 2010, see also Engel et al., 2019). For example, Lorenzi et al. (1999) asked participants to localize a train of clicks in quiet and in the presence of a white-noise masker. They investigated sound localization accuracy as a function of the frequency of the clicks, SNR, and masker location. They found that localization errors increase when noise was presented at ± 90 degrees in azimuth with respect to the source of the target signal and they suggested that this effect is due to the decreased detectability of the target signal at the ear ipsilateral to the noise (Lorenzi et al., 1999). A second finding from this literature is that increasing the number of noise sources in the environment reduces sound localization accuracy. For instance, Brungart et al. (2005) turned on different independent noise sources and they found that sound localization accuracy decreased as the number of concurrent sources increased. Finally, it has been shown that sound localization errors could be reduced by a priori knowledge about masker locations (Kopčo et al., 2010).

These previous studies are informative about the effects of noise on sound localization, and yet they focused exclusively on sound localization performance. Here we argue that at least two other aspects of sound localization experience are worth considering: first, the metacognitive evaluations related to the sound localization task and the acoustic environment itself; second, the spontaneous behaviors used by listeners when performing the task.

The first neglected aspect, metacognitive evaluations, refers to the knowledge and awareness of one’s abilities (Lai, 2011). It allows individuals to monitor their cognitive processing while doing a task or learning (Palmer et al., 2014) and they influence the individual’s ability to implement effective strategies (Zimmerman & Moylan, 2009; Schraw & Moshman, 1995; Inzlicht et al., 2021; Borkowski et al., 1987, see also Moshman, 2018). In the context of hearing research, metacognition has been extensively studied in relation to understanding speech in noise, particularly by asking participants to rate their perceived listening effort by adopting self-report procedures (e.g., Giovanelli et al., 2021; McGarrigle et al., 2019). However, much less work has examined metacognitive evaluations when listeners are engaged in a sound localization task. Rabini et al. (2020) measured perceived confidence during sound localization in quiet, while participants listened with both ears open or with one ear plugged (i.e., binaural vs. monaural listening, respectively). They found that confidence decreased in monaural listening. Moreover, they observed that sound localization accuracy and sound localization confidence can dissociate even at the single-trial level: in some trials, participants were certain but incorrect, whereas in others uncertain but correct. Valzolgher et al. (2020b) examined instead perceived effort during sound localization in quiet. They found that plugging one ear increased both localization errors and perceived effort. To the best of our knowledge, however, no study examined how metacognitive evaluations can be affected when sound localization occurs in noisy soundscapes.

The second neglected aspect concerns the spontaneous behavior that participants exploit when engaged in a sound localization task, and they are primarily exemplified by the orientation of the head. This behavior is spontaneous in the sense that it is reactive to what is happening in the environment. Previous studies on the effects of the head on sound localization have shown that spontaneous head movements improve localization skills in silence (Coudert et al., 2022; Gaveau et al., 2022; Gessa et al., 2022; Wallach, 1948). This is true even when the head movements are not spontaneous but requested or guided by the experimenter (Pastore et al., 2018; Thurlow et al., 1967). We can therefore expect that spontaneous head movements will be associated with better sound localization even in the context of noise. Again, to the best of our knowledge, no study examined how spontaneous head movements can interact with sound localization abilities in noise.

Our novel focus on sound localization experience in terms of metacognitive evaluations and spontaneous head-orientation behaviors also raises the issue of the potential interactions between these two aspects. In particular, do metacognitive evaluations play a role in triggering these spontaneous behaviors? That is, are participants more prone to make head movements when they become aware that the sound localization task is more effortful or their perception more uncertain? In the context of hearing research, this idea is supported by research findings showing that with increasing listening difficulty (e.g., with monaural hearing), participants also increase the number and extent of head movements when performing a sound localization task (Valzolgher et al., 2020a, 2020b). In addition, a positive answer to this question is predicted by the *theory of cognitive offloading*, which suggests that individuals can employ physical actions to decrease the perceived cognitive effort involved in a particular task (Dunn et al., 2019; Kool et al., 2010; Kurzban et al., 2013; Risko & Gilbert, 2016).

Interestingly, the cognitive offloading account also predicts the reverse interaction, that is spontaneous motor behavior could change the metacognitive evaluation of the ongoing task. In other words, participants could perceive the task as less demanding whenever they have adopted spontaneous motor behavior in an attempt to better cope with it. Preliminary results in this direction come from a study by Hendrikse et al. (2018), which investigated posture adjustments (head and eye-gaze movements) in normal-hearing adults during a listening-in-noise task. They observed that participants decreased the reported listening effort when they also implemented more postural adjustments. Although their study was not designed to test this hypothesis, their results suggested that behavioral strategies (e.g., spontaneous posture adjustments) could lead to reducing perceived effort during the task.

In sum, we set out to investigate the study of sound localization in noisy soundscapes with a perspective that extends beyond performance only, to also include metacognitive assessment and spontaneous behavioral strategies, as well as their interactions. To this aim, we measured sound localization accuracy, metacognitive assessments (confidence in sound position and localization effort) and spontaneous head movements while participants experienced three familiar soundscapes, a quiet nature scene, a busy traffic environment and a cocktail party context, adapted in their SNR to result of increasing difficulty. The rationale for the choice of these familiar soundscapes lies in the fact that spontaneous behaviors are a consequence of previous, episodic, experiences (Risko & Gilbert, 2016). To understand whether the metacognitive judgments people make on a given task influence the strategies they implement to carry it out, we exposed participants to each of the soundscapes prior to the actual sound localization task and collected metacognitive assessments for each of them. Particularly, we collected participants' estimated effort and self-efficacy about the task. Importantly, in this Exposure phase, the participants could not move their heads (i.e., they could not implement any spontaneous behavior that could affect the listening experience). Finally, to track head movements we used a virtual reality setup (VR). This approach also allowed us to accurately control the available visual information during the task, thus avoiding a priori visual information about the location of the noise that previous studies have shown can modulate the effects (Kopčo et al., 2010). In addition, it exploited a flexible solution to control for sound position in each trial based on the VR-guided positioning of the speaker (see Gaveau et al., 2022).

Our predictions were as follows. First, we expected that increasing difficulty of the soundscape would be associated with reduced accuracy, increased perceived effort and diminished confidence for sound localization. Second, we predicted that head-related behavioral strategies could improve localization accuracy. Third, we examined the relationship between metacognitive evaluation and head-motion behavior with two working hypotheses in mind: On the one hand, we predicted that metacognitive assessments could influence the occurrence and type of spontaneous head-motion behavior; on the other hand, we expected a decrease in perceived effort and an increase in confidence after the participant implemented head movement behaviors.

## Materials and methods

### Participants

We recruited 30 normal-hearing participants among the students of the University of Trento. We excluded one participant for her very poor sound localization ability (see Data analysis for more details). Mean age for the remaining participants was 24.4 years (SD = 3.4; 25 females). All participants had normal or corrected-to-normal vision and normal hearing (average threshold below 5.62 ± 3.66 dB HL). Hearing thresholds were measured using an audiometer (Grason Stadler GSI 17 Audiometer), testing different sound frequencies (250, 500, 1000, 2000, 4000 Hz) on the right and left ears, separately. The study was conducted in line with the Declaration of Helsinki (2013) and according to the research ethics regulations of the University of Trento. Participants read and signed informed consent before taking part in the experiment and received a certificate of participation or a gadget.

The sample size was based on the existing literature in the field of spatial hearing research that examined the effects of spontaneous head movements on sound localization using within-subject experimental designs (Gaveau et al., 2022, N = 20). Note that this is twice the size of samples used in previous studies testing sound localization in noise (e.g., Brungart et al., 2005, N = 10; Kopčo et al., 2010, N = 7).

### Apparatus

**Fig. 1:**
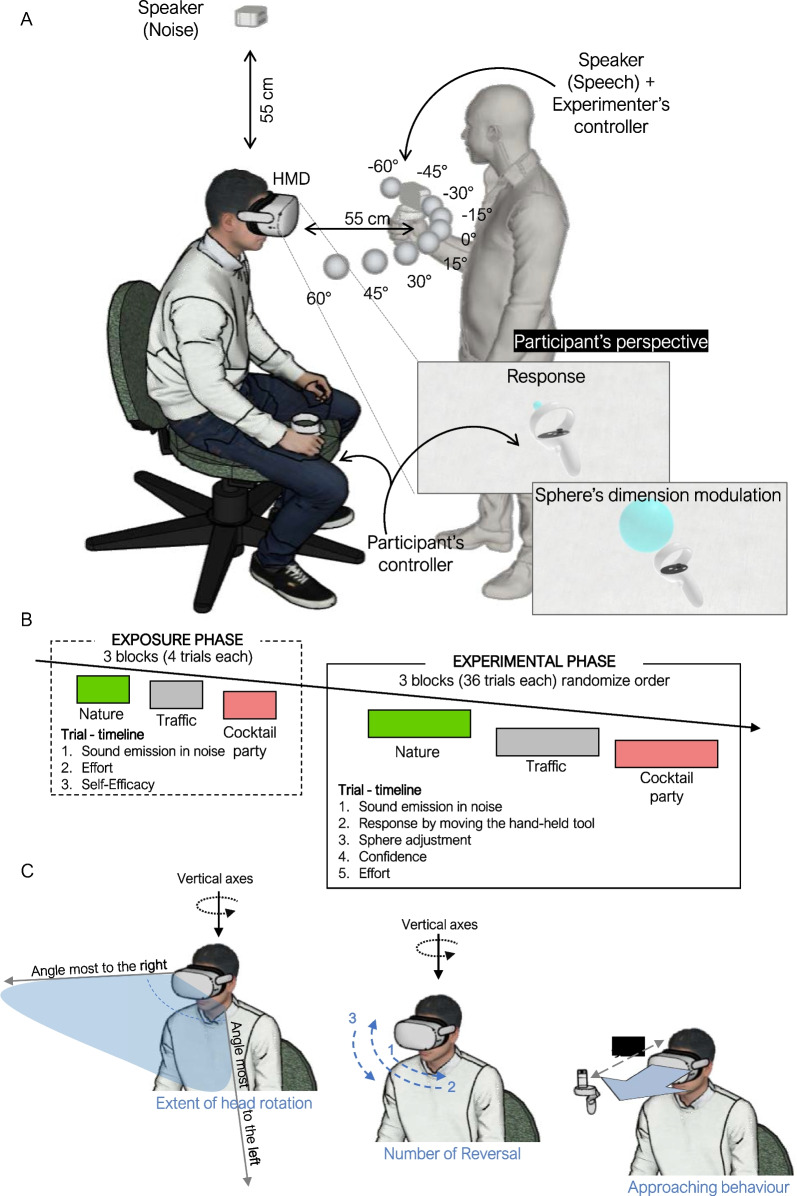
**A** Setting: schematic representation of the participant wearing the head-mounted display (HMD) and holding the virtual reality (VR) controller during the head-pointing sound localization task. The nine spheres in front of the participant indicate predetermined speaker positions (not visible in the HMD). In the bottom-right part of the figure the participant’s perspective: They were in an empty room and were instructed to locate the controller at the end of the audio track with the small sphere in the position in which they think the sound source was: **B** experimental procedure: at left, exposure phase: 3 blocks comprising a total of 12 trials. In each block, participants experienced different noisy contexts: nature, in green; traffic, in gray and cocktail party, in coral. During each trial, participants listened to target speech embedded in one of the three possible noisy contexts. At the end of each trial, they were asked to evaluate their effort and self-efficacy using a Likert scale. At right, sound localization phase: In each block, participants experienced different noisy contexts: nature, in green; traffic, in gray and cocktail party, in coral. Blocks in this phase were presented in random order. During each trial, participants listened to target speech embedded in one of the three possible noisy contexts. At the end of the sound, they were instructed to localize the source by using a handheld controller to move a light-blue sphere. They were told to adjust the size of the sphere once they were sure it covered the target position. Afterward, they had to rate how much effort and confidence they felt using a Likert scale. **C** Graphical representation of indices describing head-related behavior: extent of head rotation, number of reversals, and approaching index

The experiment was conducted in a soundproof and partially anechoic booth (Amplifon G2 × 2.5; floor area = 200 X 250 cm, height = 220 cm). Visual virtual reality (VR) and kinematic tracking was implemented using a head-mounted display (HMD; Meta Quest 2; 256 GB; resolution: 3616 × 1840; frequency: 72 Hz) and 2 controllers (one was used by participants to respond, and the other was used to track the speaker's position in real time). All stimuli were controlled and delivered using a computer (ASUS TUF Dash F15) connected to the HMD via an Oculus link cable and using homemade software developed by Unity (Unity Technologies) (see Fig. 1A).

After wearing the HMD, participants found themselves immersed in a simple virtual room, which mimicked the real room’s dimensions. To avoid localization responses induced by the presence of visual cues, the virtual scene was an empty white room. Auditory stimulation was controlled by the Unity software and delivered using two loudspeakers: The one emitting noises (i.e., nature, traffic, or cocktail party) were fixed above the participant’s head (height = 55 cm); the other speaker emitting target speech sentences were placed manually by the experimenter in predetermined spatial positions (we used only one portable speaker to deliver the target sounds). Note that the positions were determined in real-time and on a trial-by-trial basis, always considering the position of the participant’s head. The speaker held by the experimenter was associated with a controller continuously tracked by the VR system. The experimenter moved and placed the speaker according to visual instructions on a computer monitor representing a map of the sources’ positions (the current source was coloured differently), participant head (HMD) and speaker moved by the experimenter (see Valzolgher et al., 2020a or Gaveau et al., 2022 for details about this sound delivery method). Note that the software delivered both the target sound and the noise sound only when the loudspeaker reached the target position and the participant’s head was facing straight ahead. The speaker was placed in one of 9 possible positions, which changed randomly across trials. The positions were at ear level, 55 cm from the head, and in one of 9 different horizontal positions (0°, ± 15°, ± 30°, ± 45°, ± 60° with respect to the participant’s midsagittal line) (see Fig. 1A).

### Auditory stimuli

We recorded 9 short spoken sentences in Italian (i.e., “Sono proprio qui,” the Italian version of “I am right here”), uttered by both a female speaker and a male speaker. Then, we combined these sentences to create an audio target signal comprising three of them (i.e., “Sono proprio qui. Mi stai cercando? Trovami se riesci.”, in English: “I am right here. Are you looking for me? Find me if you can.”) (mean duration: 3,13 s, 44100 Hz, examples of tracks are available at osf.io/nwc6k). Each of the 9 short spoken sentences was repeated 6 times to create 18 audio target signals for both female and male speakers. The resulting 36 audio target signals were used in the 36 trials of each noise condition. In this way, we controlled for any influence of type of stimuli on the effect of noise. The sentences were recorded in a partially soundproof booth (Amplifon G2 × 2.5; floor area 200 × 250 cm, height 220 cm) using a Sennheiser E835 microphone (frequency: 40 Hz—16 kHz; sensitivity: 2,7 mV/Pa). After recording, we processed tracks in Audacity®: we cleaned audio tracks by removing background noise from the microphone with the “Noise reduction” tool, and we made the voice level of the track uniform using the “Normalize” tool.

The noise tracks were soundscapes of real environments: an outdoor nature scene (103 kbps, 48,100 Hz), a traffic scene (88 kbps, 48,100 Hz), and a cocktail party (95 kbps, 48,100 Hz; extracts of the soundscapes are available at osf.io/nwc6k). The noise tracks were pre-processed with Audacity® and adjusted to have 3 different levels. They were presented at about 55, 60 and 65 dB, respectively, as measured at ear level, while all target sentences were presented at 60 dB, as measured at ear level (measured when the target was in the central position, 0°). In this way, the three soundscapes had decreasing signal-to-noise ratios: + 5 dB when noise was from the natural environment, 0 dB when from traffic, and -5 dB when from a cocktail party. We used target speech embedded in natural soundscapes to create real-life acoustic scenarios that required different cognitive demands when doing the task and thus simulate an acoustic experience that participants may have lived in their everyday lives (i.e., studying naturalistic behavior, see Krakauer et al., 2017). The logic behind the choice of these stimuli is related to the objective of the experiment: Our aim was not an accurate analysis of the acoustic effects of localizing a spoken stimulus in noise, but rather to study spontaneous behaviors and movements of the subject which may be promoted by experiencing acoustic naturalistic scenarios. To let participants experience the soundscape, the noise started 1 s before and ended 1 s after the target speech sentence presentation (mean duration of each acoustic experience per trial: 5.13 s).

### Procedure

The entire experimental session lasted about 1 h, and participants were seated on a rotating, armless chair with no chin rest. First, participants were asked to wear the HMD and were then instructed on the procedure.

In the first part of the experiment (Exposure phase), participants were exposed to each soundscape in a fixed order of increasing complexity (i.e., nature, traffic and cocktail party; 12 trials in total, i.e., 4 exposure trials for each noise condition). During the exposure phase, the target sentences were presented from 4 different locations (ear level, 55 cm of distance and varying from ± 20° and ± 40° along the horizontal dimension), and participants were only asked to report metacognitive estimates of the situation. In every noise condition, the 4 locations were repeated 1 time. Specifically, they were asked to estimate sound localization effort and self-efficacy in doing the task (“*How much effort would you require to localize target speech source in this situation?”* from 1 = none to 6 = much; “*How well would you be able to localize the target speech source?”* from 1 = not at all to 6 = very well. The original questions were in Italian: “Quanto sforzo ti richiederebbe localizzare la fonte sonora? and “Quanto ti senti in grado di localizzare la fonte sonora?”). Note that during the exposure phase, no head movements were allowed (Fig. 1B). The rationale of the exposure phase was to collect participants' estimated effort and self-efficacy about the specific task. Thus, to give participants an idea of the type of the task we exposed them to a brief experience of it (4 trials). Plus, to give participants the possibility to adjust their evaluations, we exposed them to an increasingly complex scenario: The order of noise presentation in the exposure phase was fixed: nature, traffic and cocktail party.

During the second part of the experiment (sound localization phase), the participants completed 108 trials divided into 3 blocks, one for each noise condition (36 trials per block). Half of the target sentences were spoken by a female voice and the remaining half by a male voice (divided equally in each block). They were emitted from 9 possible azimuth positions (Fig. 1A), which were repeated for 4 times per block. The block order (noise) was counterbalanced among participants. To help participants direct their heads at the beginning of each trial, a cross was presented to the participant, and it moved accordingly with the participant’s head movement. It turned green when placed in the correct starting position by facing straight ahead. Once sound onset began, participants were free to move their heads and trunks if they wanted. After presenting the auditory stimulation, participants were asked to move the controller to the perceived location of the sound and to validate their response by pressing a button on the controller. To help them, the controller was represented in the virtual room, and a blue sphere was positioned above the controller to indicate precisely where they were positioning their response.

As a further measure of certainty in doing the sound localization task, after validating the response, participants were asked to change the size of the blue sphere by moving the controller in their hand to set the radius that they were sure would include the correct position of the sound. When satisfied, they could validate their response to confirm the sphere size. Participants were instructed to maintain the sphere with a modest dimension (i.e., the diameter before manipulating it was about 2 cm) if they were sure that the source was precisely in the position in which they placed the sphere. On the contrary, they had to increase the sphere dimension if they were not confident about the position in which they localized the sound source. In other words, sphere size represents the portion of space in which participants thought that the source could be positioned. We registered sphere diameters (cm) and analyzed them as an index of uncertainty. We added it to capture a spatial dimension of the feeling of uncertainty directly related to the goal of the task: defining spatial position of sounds. By introducing this measure, we added a secondary aim to the study: to observe whether this measure could be modulated by the proposed manipulations and could help describe the metacognitive/evaluative processes related to acoustic space perception. A similar procedure was recently adopted by Fassold et al. (2023) to measure confidence. They involved participants in a reaching-toward-visually targets task and asked them to change the size of a circle (2d) centered on the reach-target location to determine their level of confidence. As for the here-proposed sphere, a larger circle reflects lower confidence. Next, participants were asked to judge their confidence about their response *(“Are you sure that the sound was emitted by the position you selected?”* from 1 = not sure to 6 = sure; the original version: “Quanto sei sicuro di aver localizzato correttamente il suono?”) and their perceived effort during the trial *(“How much effort did the task require?”* from 1 = none to 6 = much; the original version: “Quanto sforzo ti ha richiesto il compito?”). At the end of the session, the experimenter provided complete details about the study and its purpose.

### Data analysis

To study performance, we measured the absolute error along the horizontal dimension. This was obtained by calculating the discrepancy between the position of the speaker and participant responses for each trial in absolute values. Furthermore, we examined the standard deviation of signed error values grouped according to each speaker position to assess the variability in participants' responses, which serves as a measure of precision.

To describe head movements, we used the data recorded by the head-mounted VR. Specifically, the headset we used (a Meta Quest 2) relies on a combination of onboard cameras and sensor technology to precisely track the user's head and hand movements. The cameras capture images of the user's surroundings and controllers, while computer vision algorithms analyses these images to identify and track specific visual features. For example, the controllers have infrared LEDs that are seen by the cameras and recognized by the algorithms. These algorithms then calculate the user's head and hands positions and orientations in real time. This tracking system is referred to as an "inside-out tracking system", which eliminates the need for external sensors that other headsets have. Participants were allowed to move their heads and trunks as they wanted during the sound localization phase. However, the kinematic tracker registered exclusively the head rotation and the head position in the space. Thus, we are not able to disambiguate head vs. trunk movements. We focused on the head movements only, while being aware that moving the trunk could also contribute to performing this type of movement as naturally happens when people turn around. We consider the entire time window of audio stimulation (from the beginning of the noise to the end of the audio). The rationale was to consider the entire head-related activity of participants during the acoustic experience. We were not interested in studying in-depth the effect of movements on SNR levels, but we were focused on the general motor activation that participants could implement in various listening contexts.

The three indices that we adopted to describe head movement behavior are rendered graphically in Fig. 1C. They represent the more relevant behavioral strategy, considering our sound localization task, as they can change binaural cues on the two ears (Kato et al., 2003; McAnally & Martin, 2014). Precisely, we measured the number of reversals during one trial. For this specific variable, we considered only movements for those reversals wider than 5 degrees to exclude micro-postural movements not related to the task and to measure movements that were implemented more actively and intentionally by participants, as they might be linked to metacognitive evaluation. However, it could be that smaller movements play a role in favoring sound localization (see, for instance, McLachlan et al., 2023), but considering them in this context was beyond the scope of this study. Plus, we calculated the extent of head rotations around the vertical axis (i.e., the sum of the absolute value of the rightward and leftward head rotation extremity reached by the participant in each trial, see Fig. 1C). This measure reflected the amount of the space explored by rotating the head. Furthermore, we calculated a measure of approaching behavior. In calculating this measure, we did not consider head rotation around the vertical axis, we considered the values in centimeters of the minimum distance between the head position and the speaker emitting the sound reached during the trial. Note that the starting distance at the beginning of the trial was about 55 cm, but participants were allowed to move naturally during the trial. Thus, they could also move their heads to approach the space in front of them and thus the target speaker (Fig. 1C).

Linear and generalized linear mixed-effect models, ANOVAs and non-parametric tests (Spearman correlation) were used for statistical analyses. Statistical analyses were run using R (version 1.0.143). For the linear and generalized linear mixed-effect model, we used the R-packages emmean, lme4, and lmerTest in R Studio (Bates et al., 2014; Fox & Weisberg, 2020). When performing linear and generalized linear mixed-effect modeling, we first considered the distribution of the dependent variable and we opted to either log-transforming the data or adopt general mixed-effect models (gamma or Poisson distributions) in case of evidently skewed distribution. Second, we observed the distribution of the model’s residuals to visually check the assumption of their normal distribution. Third, when we have an appropriate model, we run likelihood ratio tests by using ANOVA function of the car package in R to obtain deviance tables and describe models’ results in terms of main effect and interactions. Contrasts were run by using function emmeans in R which uses Tukey adjustment by default. See the Result section and the code available in the OSF for further information (see direct link below). Participant 20 was removed by the analysis because she was clearly an outlier when considering absolute errors (very poor sound localization ability, i.e., 58° absolute error in the cocktail party condition). Furthermore, the head data of Participant 12 was not considered due to technical problems with the kinematics tracking. For the remaining participants, 0.5% (for performance) and 0.4% (for head data) of trials were removed from analyses due to a lack of data (e.g., technical problems such as the disconnection of cables during the trial or software bugs) or errors in sound delivery (e.g., the experimenter erroneously moved the speaker during sound emission). During the exposure phase, 2.9% of the values for effort were not registered due to participant error in validating responses, while the self-efficacy of participant 1 was not collected. Data and code can be retrieved from osf.io/nwc6k.

## Results

### Effects of soundscapes on localization performance, metacognitive evaluations and head movements

**Fig. 2:**
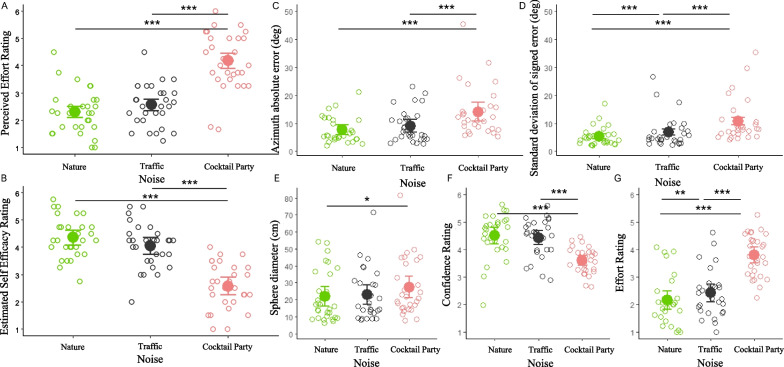
Effect of noise: **A** and **B** Participant self-evaluations (effort and self-efficacy) during the exposure phase, as a function of noise; **C** and **D** Participant absolute error and standard deviation of signed error as a function of noise. **E**, **F** and **G** Sphere diameter, confidence and effort reported during sound localization phase as a function of noise. The nature noise condition is shown in green; traffic, in gray and cocktail party, in coral. Filled circles represent average for each subject (but note that the LME-based analyses used data in each trial with participant-specific random effects). Error bars represent confidence intervals at 95% (calculated by using the function summary SE in R)

First of all, we analyzed the effect of soundscape on perceived effort during the exposure phase, when no actual localization was required and head movements were not allowed. We enter the perceived effort rating into a linear mixed-effect model with noise as a fixed effect. We fitted random intercept for participants and random slope by participants for the noise factor. We observed a main effect of noise (*X*^*2*^ (2) = 99.61, *p* < 0.001), revealing that the expected sound localization effort was higher in the cocktail party (2.6 ± 0.8) compared to both the traffic (2.5 ± 0.8, *t* = 9.17, *p* < 0.001) and nature noise conditions (2.3 ± 0.8, *t* = 8.75, *p* < 0.001), but no differences emerged between nature noise and traffic conditions (*t* = 1.46, *p* = 0.33) (Fig. 2A). A similar analysis was run by considering estimated sound localization self-efficacy. We observed a main effect of noise (*X*^*2*^ (2) = 133.46, *p* < 0.001), revealing that sound localization self-efficacy was lower for the cocktail party (4.2 ± 1.1) compared to both the traffic (4.1 ± 0.8, *t* = 9.89, *p* < 0.001) and nature noise conditions (4.4 ± 0.7, *t* = 10.93, *p* < 0.001), but no differences emerged between nature noise and traffic conditions (*t* = 2.28, *p* = 0.07) (Fig. 2B). We computed for each participant for each noise condition a mean value and run Spearman correlations. These analyses showed that the higher the perceived effort, the lower the self-efficacy in all noise conditions (*p* ≤ 0.001 for all).

Then, we analyzed participants' performance during the sound localization phase. We enter the absolute error into a linear mixed-effect model with noise and trial number as a fixed effect. We fitted random intercept for participants and separated random slopes by participants for the noise and for the trial number factor. The choice was determined by the fact that the model did not converge if considered the highest-order combination of within-subject factors. We observed a main effect of noise (*X*^*2*^ (2) = 45.71, *p* < 0.001), revealing that absolute error was larger during the cocktail party (14.1° ± 9.0°) compared to the traffic (9.1° ± 5.8°, *t* = 5.16, *p* < 0.001) and nature noise conditions (7.7° ± 4.7, *t* = 6.62, *p* < 0.001), but no differences emerged between nature noise and traffic conditions (*t* = 1.64, *p* = 0.25) (Fig. 2C). Plus, we observed a main effect of the trial number (*X*^*2*^ (1) = 4.45, *p* = 0.03) revealing that absolute errors decreased as a function of trial repetition (slope estimated by the model = -0.04). We did not find any effect of interaction between trial number and noise (*X*^*2*^ (2) = 5.71, *p* = 0.06). A similar analysis was run on standard deviation values of signed error by considering noise as a fixed effect and intercept for participants and slope by participants for the noise factor as random effects. We found a main effect of noise (*X*^*2*^ (2) = 101.14, p < 0.001), revealing that standard deviation of signed error was larger during the cocktail party (10.8° ± 7.6°) compared to the traffic (6.8° ± 5.7°, *t* = 6.06, *p* < 0.001) and nature noise conditions (5.3° ± 3.3°, *t* = 10.05, *p* < 0.001). Plus, a difference emerged between nature noise and traffic conditions (*t* = 2.78, *p* = 0.03) (Fig. 2D).

We proceeded by analyzing the metacognitive measures during the sound localization phase. We entered the sphere diameter into a linear mixed-effect model with noise and trial number as a fixed effect. We fitted random intercepts for participants and random slopes by considering the highest-order combination of within-subject factors. We observed a main effect of noise (*X*^*2*^ (2) = 9.55, *p* = 0.008, Fig. 2E), revealing that the sphere diameter was larger during the cocktail party (27.3 cm ± 16.6 cm) compared to nature noise conditions (21.9 cm ± 15.1 cm, *t* = 3.08, *p* = 0.01). However, we did not find differences between traffic (23.0 cm ± 14.8 cm) and nature (*t* = 1.76, *p* = 0.20) and traffic and cocktail party (*t* = 2.31, *p* = 0.07). No main effect of trial number or interaction emerged (*ps* > 0.67). Similar analyses were run by considering confidence and effort as dependent variables. We observed a main effect of noise on confidence (*X*^*2*^ (2) = 80.16, *p* < 0.001) revealing that confidence rating was lower during the cocktail party (3.6 ± 0.5) compared to the traffic (4.4 ± 0.7, *t* = 8.61, *p* < 0.001) and nature noise conditions (4.5 ± 0.8, *t* = 7.56, *p* < 0.001). No difference emerged between traffic and nature (t = 0.80, *p* = 0.37) (Fig. 2F). We also documented a main effect of trial number (*X*^*2*^ (2) = 15.50, *p* < 0.001) revealing that confidence increased across trials (slope estimated by the model = 0.08). No interaction between trial number and noise was observed (*p* = 0.33). Analysis on effort showed a main effect of noise (*X*^*2*^ (2) = 107.94, *p* < 0.001). Effort increased during the cocktail party (3.8 ± 0.8) compared to the traffic (2.4 ± 0.9). In both cocktail party and traffic conditions, effort was higher as compared to nature noise condition (2.2 ± 0.9, *p* < 0.009 for all contrasts) (Fig. 2G). We did not observe any effect of trial number or interaction (*p* > 0.14).

During the sound localization phase, we run a general linear mixed effect model (family = poisson) by considering the number of reversals as dependent variable and noise and trial number as fixed effects. We fitted random intercepts for participants and random slopes by considering the highest-order combination of within-subject factors. We did not find any main effect or interaction (*p* > 0.14 for all). A similar model (family = gamma (link = log)) was run by considering the extent of head rotation as dependent variable and we did not document any effect (*p* > 0.23 for all). Finally, another mixed effect model (family = gamma (link = log)) was run by considering the approaching index, again without finding any effect of noise and trial number or interaction (*p* > 0.22 for all).

### Relationship between variables

#### Head movements and performance

**Fig. 3:**
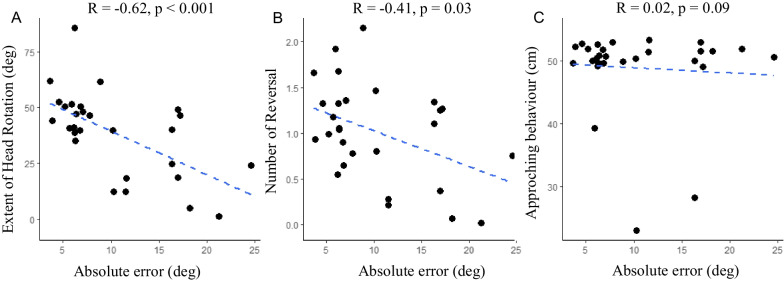
Spearman correlation between head-related behavior (mean number of reversals (**A**); mean extent of head rotation (**B**) and mean approaching index (**C**)) and performance (mean absolute error), irrespective of noise type. Each point showed average values calculated for each participant. Note that the approaching index refers to the values in centimeters of the minimum distance between the head position and the speaker emitting the sound reached during the trial. The starting distance at the beginning of the trial was about 55 cm, but participants were allowed to move naturally during the trial. Small values ​​indicate that the participant came closer to the target

We considered the correlation between the three variables describing head movements and the two variables describing performance. We computed for each participant a mean value by collapsing all the noise conditions, and run Spearman correlations. We found that a relation between head-related behavior and performance emerged irrespective of soundscape type. The greater the extent of head rotation (Spearman *R* = -0.62, *p* < 0001, Fig. 3A) or the higher the number of reversals (Spearman *R* = -0.42, *p* = 0.03, Fig. 3B), the lower the absolute error. Likewise, the greater the extent of head rotation, the lower the standard deviation of signed error (Spearman *R* = -0.48, *p* = 0.01). We found no correlations between the amount of approaching behavior and the two performance measures (absolute error and standard deviation of signed error) (*p* > 0.21 for all, see Fig. 3C). In Table 1 we reported for completeness all the correlations. Note that we did not correct the significance for multiple correlations.

**Table 1:** Spearman correlations between head movement variables (number of reversals, extent of head rotation and approaching behavior) and performance variables (Absolute error, Standard deviation of signed error)

	Number of reversals	Extent of head rotation	Approaching behavior	Absolute error
Number of reversals				
Extent of head rotation	0.68***			
Approaching behavior	− 0.42*	− 0.01		
Absolute error	− 0.42*	− 0.62***	0.02	
Standard deviation of signed error	− 0.21	− 0.48*	− 0.09	0.87***

#### Head movements and metacognitive evaluations

We first examined the relationship between metacognitive evaluations measured during the exposure phase (i.e., perspective judgments expressed before performing the task) and head movements during the sound localization phase. This allowed us to investigate if sound localization effort or estimated self-efficacy in sound localization associated with each of the noise conditions had an impact on the head movement behavior observed later in the task. However, no such relationship reached significance (*p* > 0.24 for all, except for the correlation between approaching behavior and self-efficacy in the nature condition and between effort and number of reversals in the traffic condition, which both approached significance, *p* = 0.06 and 0.07 respectively) (see Additional file 1: Table S1).

Having established that the metacognitive judgment expressed in each of the noise conditions during the exposure phase did not influence subsequent head movements, we turned to examine if a relationship between metacognition and head movements could exist when participants were engaged in the sound localization task—i.e., when both measures were obtained during the sound localization phase. We computed for each participant a mean value by collapsing all the noise conditions, and run Spearman correlations. No correlation emerged between the metacognitive evaluations measured before and during the task, indicating that participants were rating two different metacognitive states during the Exposure and sound localization phases (all correlations are reported in Table 2).

**Table 2:** Spearman correlations between metacognitive measures collected during the exposure phase (estimated effort and estimated self-efficacy) and during the sound localization phases (perceived effort and confidence)

	Estimated effort	Estimated Self-efficacy	Effort	Confidence
Estimated effort				
Estimated self-efficacy	− 0.60***			
Effort	0.03	− 0.22		
Confidence	− 0.04	0.45	− 0.55**	

**Fig. 4:**
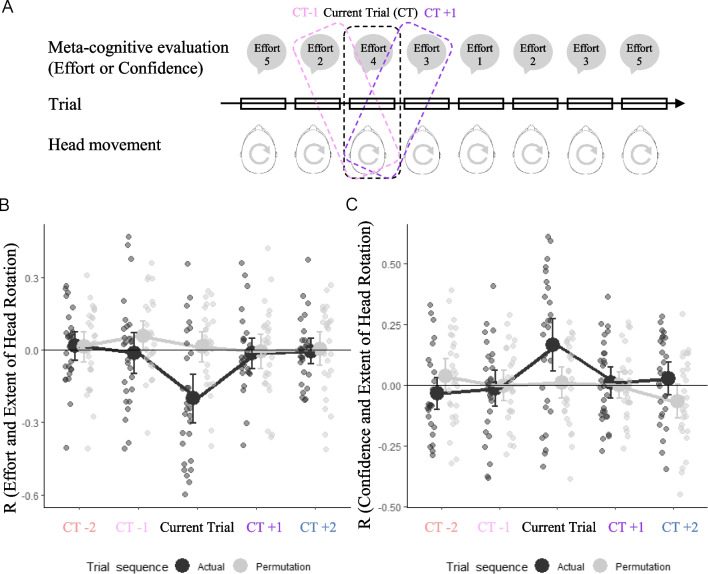
**A** Graphical representation of the computation of the correlation index between metacognitive evaluations and head-related behaviors across trials. **B** Correlation values obtained considering effort in the actual vs. permutation sequences of trials as a function of the trial time (CT − 2, CT − 1, CT, CT + 1, CT + 2). **C** Correlation values obtained considering confidence in the actual vs. permutation sequences of trials as a function of trial time (CT − 2, CT − 1, CT, CT + 1, CT + 2)

To further examine the relationship between metacognition and head movements during the sound localization phase, we computed correlation indices between metacognitive evaluations and head movements during the sound localization phase by considering the values recorded during the same trial (current trial), as illustrated in Fig. 4A. Because the metacognitive measure was always computed at the end of the trial (i.e., after any head movement already occurred) correlation at the current trial (CT) indicates to what extent performing a head movement influenced the immediate metacognitive judgment. Furthermore, we computed correlation indices by considering metacognitive evaluation values reported by the participants up to two trials after (i.e., CT + 1 and CT + 2) and up to two trials before (i.e., CT− 1 and − 2) each trial. Correlations measured for CT + 1 and CT + 2 test for the hypothesis that the effect of a head movement influences metacognitive judgment in subsequent trials (trial + 1 or trial + 2 with respect to when the head movement occurred). On the contrary, correlations measured for CT− 1 and CT− 2 test for the hypothesis that the metacognitive judgments expressed in the trials preceding the head movement (i.e., trial − 1 and trial − 2) influence its occurrence. We extracted these correlation values as a function of trial time separately for each metacognitive variable (i.e., effort and confidence), using head rotation extent as an indicator of head movement. The rationale for running this exploratory analysis on this dependent variable is that head rotation extent describes participants' tendency to explore the acoustic space from a global perspective and it was documented to be relevant to facilitate sound localization (see Valzolgher et al., 2020b). Because the cocktail party noise condition was perceived by the participants as the more effortful and was judged as the noise condition which evoked less confidence, we limited this analysis to this challenging listening condition. Importantly, we contrasted these correlation values obtained from the actual data with the correlations in data from the permutated sequence of head movement measures across trials (reported in light gray in Fig. 4B, C). The permutated sequence of head movement was calculated separately for each participant.

We entered correlation values in the actual versus permutation sequences of trials in a repeated measures ANOVA with data (actual vs. permutation) and trial time (CT − 2, CT − 1, CT, CT + 1, CT + 2) as independent variables. As shown in Fig. 4B, we observed an interaction between trial time and data when considering effort as a metacognitive measure (*F*(4,108) = 495, *p* = 0.001). This interaction was determined by the difference between original and permuted data when the trial is the current one (*t*(27) = 4.14, *p* < 0.001), suggesting that a greater extent of head rotation reduced the perceived effort for the current trial (i.e., the two measures were collected within the same trial) (Fig. 4B). Similar results were observed considering confidence, in which, for the current trial, the confidence increased as the extent of head rotation increased (*F*(4,108) = 3.71, *p* = 0.007; *t*(27) = 2.85, *p* = 0.008) (Fig. 4C).

To further explore this issue, we run a linear mixed-effect model. We enter the CT (current trial) effort reported during the sound localization phase as dependent variable and consider noise and extent of head rotation as a fixed effect. We fitted random intercept for participants and random slopes by participants for the noise factor. We observed a main effect of noise (*X*^*2*^ (2) = 98.95, *p* < 0.001) revealing that effort rating was higher during the cocktail party compared to the traffic and nature and in the traffic as compared to nature (*p* > 0.01 for all). Plus, we observed a main effect of the extent of head rotation (*X*^*2*^ (1) = 32.51, *p* < 0.001) revealing that wider extent of head rotation determined a lower effort rating (estimated slope = -0.003743). Interestingly, we also found a significant interaction between head rotation extent and noise (*X*^*2*^ (2) = 28.53, *p* < 0.001). This result suggested that even if in all noise conditions higher values of the extent of head rotation determine a reduction in the effort this influence was stronger during the cocktail party as compared to traffic (*t* = 4.43, *p* < 0.001) and nature (*t* = 4.89, *p* < 0.001), while no difference has been observed between traffic and nature (*t* = 0.40, *p* = 0.92). Then, we run another linear model by entering the CT confidence reported during the sound localization phase as dependent variable and consider noise and extent of head rotation as a fixed effect. We fitted random intercept for participants and random slopes by participants for the noise factor. We observed a main effect of noise (*X*^*2*^ (2) = 87.64, *p* < 0.001) revealing that the confidence rating was lower during the cocktail party compared to the traffic (*t* = 7.89, *p* < 0.001) and nature (*t* = 6.87, *p* < 0.001), while no difference emerged between traffic and nature (*t* = 0.75, *p* = 0.74). Plus, we observed a main effect of the extent of head rotation (*X*^*2*^ (2) = 37.79, *p* < 0.001) revealing that wider extent of head rotation determined a higher confidence rating (estimated slope = 0.003932). Interestingly, we also found a significant interaction between head rotation extent and noise (*X*^*2*^ (2) = 28.96, *p* < 0.001). This result suggested that even if in all noise conditions higher values of the extent of head rotation determine an increase in the confidence, this influence was stronger during the cocktail party as compared to traffic (t = 3.68, p < 0.001) and nature (*t* = 5.26, *p* < 0.001), while no difference has been observed between traffic and nature (*t* = 1.63, *p* = 0.24).

## Discussion

In this work, we aimed to study the effects of noisy soundscapes on sound localization considering performance, metacognitive assessment and spontaneous behavioral strategies. Three main findings emerged. First, the noisy soundscapes affected both performance errors and metacognitive evaluations. Participants increased their localization errors and reduced their precision (i.e., increased standard deviation of signed error) as the complexity of the noisy environment increased. These changes in performance were accompanied by reported increased effort, decreased confidence and increased uncertainty (as measured by sphere dimension) in sound localization. However, we did not find any effect of soundscape on head-related behavior. The second main finding was that head movements played a beneficial role in reducing sound localization errors in noisy conditions. Participants who exhibited more head movements and explored a greater spatial extent made fewer errors in sound localization. Third, head movements performed during the sound localization task were not influenced by the metacognitive evaluations expressed before task execution (i.e., during the exposure phase). However, we found preliminary evidence that, during task execution, head movements reduced perceived effort and increased confidence levels of the participants. In the next paragraphs, we discuss each of these findings in turn.

### Assessing the effects of noisy soundscapes beyond sound localization performance

Our first finding is consistent with previous studies that have shown decreased localization ability as the noise increased (Brungart et al., 2005; Kopčo et al., 2010; Lorenzi et al., 1999). It is important to note that, differently from previous works, we did not directly compare sound localization in quiet vs. noise. We analyzed instead the effect of noise on sound localization by modulating it along a continuum that varied from a real-life natural condition mimicking a silent and acoustically poor environment (i.e., nature noises) to a richer one (i.e., cocktail party).

Interestingly, we found an effect of noise on subjective evaluations provided by participants: as the noise increased, the localization task was judged more effortful and participants reduced confidence. This is not the first study that investigated these aspects in the field of acoustic perception. In previous hearing-in-noise research, metacognitive measures, including the assessment of perceived effort, have been already incorporated. A notable example is the study of Giovanelli et al. (2021) who measured performance, confidence, listening effort and metacognitive monitoring (the ability to adapt self-judgments to actual performance) in classical hearing-in-noise task in which participants were asked to recognize a sentence pronounced by a target speaker while other speakers were simultaneously pronouncing other sentences. They observed that increasing the number of distracting speakers as well as hiding the talkers behind a screen or concealing their lips via a face mask led to lower performance, lower confidence scores, and increased perceived effort (see also Van Den Tillaart-Haverkate et al., 2017). Moreover, measuring listening effort is particularly relevant when studying individuals with hearing impairments, as they often report feeling fatigued during listening tasks. This measure goes beyond mere performance metrics and captures additional dimensions of the listening experience (Peelle, 2018; Pichora-Fuller et al., 2016). However, when describing the sound localization experience, the feeling of fatigue and confidence has not often been asked to participants. An interesting exception was the study of Rabini et al. (2020), who measured perceived confidence in a sound localization task in quiet. They observed the effect of manipulating the auditory cues by plugging one ear of participants and observed that monaural listening decreased perceived confidence. As far as we know, Rabini et al.’ study is one of the few studies that have included metacognitive evaluations in a sound localization task. In line with this result, with the present study, we started to include the measurement of such dependent variables in the context of sound localization in noise experience. By including metacognitive measures, we aimed to capture the complexity of cognitive processes involved in mapping the acoustic space as they can be modulated by the background noise.

In this study, we also introduced a novel measure of uncertainty. Specifically, we recorded the diameters of the spheres that participants were instructed to draw after each sound localization response, and we analyzed these measurements as an indicator of uncertainty. In line with the recent work by Fassold et al. (2023), we incorporated this measure to capture a spatial dimension of the feeling of uncertainty directly relevant to the task's objective: Defining the spatial position of sounds. Interestingly, sphere diameters increased more during the cocktail party condition compared to the traffic and nature noise conditions. This finding indicates that as the soundscape increased in level and complexity, participants allocated a larger area of space to the perceived source. Future studies could leverage further on the virtual reality potentials to introduce similar response measures, which can contribute to describing the metacognitive aspects involved in the perceptual experience.

A further aspect worth considering concerns the nature of the difference between the three soundscapes we proposed. Throughout the manuscript, we used the term complexity to describe the three acoustic situations. In these cases, complexity was determined both by the level of SNR, which varied in the three conditions, and by the content of each noise track, which contained nature sounds or traffic sounds or background noise composed by overlapping voices of people in the cocktail party situation. The rationale that guided our choice was to create everyday life soundscapes able to evoke a different range of familiar acoustic experiences. Each experience could have been evaluated differently by participants and thus could have evoked various behaviors. Yet, this solution does not allow us to discern between the effects of SNR and the type of sounds contributing to the soundscapes. Indeed, note that SNR and type of background noise can also interact in their influence on speech perception and effort. Interestingly, speech intelligibility might have been affected by the noise type considering both its informational and energetic components. Thus, it might have affected the related experience of effort in individuating both the target speech and its position (Krueger et al., 2017). However, note that participants were not asked to identify the speech messages, but they served only as targets of the localisation task and were always intelligible. Thus, it was most likely that the effort related to sound localization experience even because the question, as well as the instructing, were focused on this task. In future works, it would be interesting to discriminate if the effects on metacognitive judgments and head-orienting behavior we describe are differently affected by SNR and type of sound.

### Spontaneous head movements improve sound localization in noise

The number of reversals, the extent of head rotation and the approaching behavior did not change as a function of soundscapes, and yet these behaviors clearly affected performance. One notable observation of the present work is that participants who exhibited more head movements and who explored a greater spatial extent made smaller localization errors. This result is in line with the growing body of research that highlights the benefits of head movements in reducing sound localization errors (Coudert et al., 2022; Gaveau et al., 2022; Gessa et al., 2022; Pastore et al., 2018; Thurlow et al., 1967). Plus, it extended the observations of this benefit toward listening while immersed in common noisy contexts. This result has important implications from an applied perspective, as it emphasizes the significance of listening by assuming an active attitude (e.g., by moving head and body) in everyday situations to enhance people's ability to process acoustic space. To further advance our understanding, future studies should investigate the role of active listening in noisy and real-life valid contexts, particularly by examining individuals with hearing impairments. By extending previous findings to clinical settings, such research can provide valuable insights for improving sound localization abilities and enhancing auditory experiences for individuals with hearing deficits (Coudert et al., 2022; Gessa et al., 2022).

We did not find any effect of noisy context on participants' head-related behavior. This could be because participants were not explicitly instructed to move and, hence, their movements were completely spontaneous. It may have been different to explicitly suggest functional listening movements to the participant as proposed in previous studies (Pastore et al., 2018; Thurlow et al., 1967). Plus, since they were not immersed in a visual environment, they did not have any visual reference of possible target to approach. This aspect could also contribute to refrain them to move closer to the sound. Future studies are needed to deepen these aspects by paying attention to the distinction between spontaneous and induced movements. Plus, future studies could propose different or more challenging noisy contexts, thus contributing to further exploring the effect of the type of background noise on spontaneous behaviors.

### Metacognitive judgments and head movements: preliminary evidence of interactions

Our study also constitutes a first attempt toward the study of the link between the spontaneous head movements when localizing sounds and the metacognitive evaluations of the participants. Recall that in our experimental design, we distinguished two phases. In the exposure phase, we collected metacognitive evaluations before participants engaged in the task and they could not implement any head movement. Instead, in the sound localization phase, we collected metacognitive evaluations while participants performed the task and were free to move their heads. Our results showed no relationship between the metacognitive evaluations expressed during the exposure phase and the subsequent head behavior measured in the sound localization phase. Hence, perspective metacognitive evaluations of effort and self-efficacy did not seem to drive subsequent spontaneous head movements. Instead, we found initial evidence of a relationship between metacognitive judgments and head movement when participants were engaged in the task.

The absence of a relationship between evaluations from the exposure phase and the behavior implemented during the sound localization phase conflicts with the hypothesis that the estimated cognitive effort required by a specific task determines the physical actions implemented in a related context (Kool et al., 2010; Kurzban et al., 2013; Risko & Gilbert, 2016; Risko et al., 2014). In our paradigm, however, several aspects may have impeded the observation of such a relationship. Asking participants not to move during the exposure phase may have influenced their evaluations of the estimated effort and self-efficacy. In addition, the duration of the exposure phase in our study, which consisted of four trials per condition, may not have allowed participants enough time to fully appreciate the metacognitive state associated with the particular task. The absence of a correlation between metacognitive judgments collected during the exposure phase (a priori judgments) and those obtained during the sound localization phase may indicate that individuals' subjective evaluations of their cognitive experiences (e.g., effort) appear to be subject to substantial modification as they transition from anticipatory assessments to real-time task engagement. This implies that the foreknowledge of a given task's cognitive demands may be inadequate for establishing a consistent representation of the ensuing effort requirements. Consequently, the act of task execution significantly influences the concurrent metacognitive evaluations, at least in this particular task. Accordingly, establishing a direct relationship between the judgments provided during the exposure phase and the head-related behaviors observed during the actual implementation of the task becomes unlikely.

Yet, preliminary evidence of a relationship between head movements and metacognitive evaluations emerged while participants performed the sound localization task. We found that greater extent of head rotation reduced the perceived effort for the current trial. This result emerged also when considering confidence: Perceived confidence increased as the extent of head rotation increased. Interestingly, the fact that wider space explored by rotating the head (i.e., wider extent of head rotation) led to a reduction in perceived effort and an increase in confidence was confirmed by our results adopting different statistical analysis strategies. Plus, it was observed that this relationship was even stronger when the soundscape was the most complex one (i.e., cocktail party). This suggests that in particularly challenging listening conditions, greater motor activation leads to even more positive consequences on our subjective evaluation (i.e., perceived effort and confidence) of the perceptual experience.

Previous findings in this direction have been observed in tasks related to hearing experience in noise. Hendrikse et al. (2018) showed that the experimental conditions in which participants implemented more postural adjustments were the ones in which they reported higher listening effort. More recently, we found (Gessa et al., under review) that trunk and head postural adjustments reduce listening effort in a speech-in-noise task. The results of the present study expand these previous findings by showing that the consequences of implementing behavioral strategy on metacognitive evaluation (e.g., effort or confidence) can also extend to spatial hearing. Plus, they prompt us to reflect on the role of active experience in the processing of metacognitive evaluations: being actively engaged in the task can, per se, contribute to different effort and confidence evaluations, compared to static and passive estimations. These findings push research to investigate more the cognitive and metacognitive processes that are related to behavioral strategies within and beyond acoustic perception and suggest further investigating the role of actively being involved in a certain task in metacognitive processing (see also Thomas et al., 2022).

In part of the analyses we conducted in this study, we tried to assess the influence of the temporal progression (i.e., trials) on performance, behavioral, and metacognitive aspects of sound localization experience. We found the effect of trial number only on absolute error and confidence. We showed that absolute error decreased and confidence increased across trial repetition. Both observations suggest that participants improved across time in the sound localisation task. However, we did not observe any change across trials when considering head movements. Our rationale stems from the assumption that studying the evolution of these variables over time represents a pivotal way to characterize their associations. It is worth acknowledging that the utilization of 36 trials per block in this study might be a limitation, as it could be deemed insufficient for capturing a pronounced temporal evolution. As we look toward future investigations, they may benefit from the insights resulting from this initial exploration and place an increasing emphasis on the nuanced examination of temporal dynamics in these domains.

## Conclusions

In conclusion, our study has provided novel findings on the impact of noisy soundscapes on the sound localization experience, extending the perspective to include performance, metacognitive evaluations and head movements. Plus, it provided new insights into the interactions between behavioral strategies and metacognitive evaluations in the context of spatial hearing. From an applied standpoint, gaining insight into this relationship could offer guidance to individuals, regardless of their hearing ability, in regulating their behavior in alignment with their goals and internal states and effectively adapting it when listening in noisy environments in everyday life.

### Supplementary Information

**Additional file 1. Supplementary Table 1.** Spearman correlations between head movements variables (Number of reversals, Space explored with the head and Approaching behaviour) collected during the sound localization phase and the metacognitive variables of estimated effort and self-efficacy collected in the exposure phase. Values are not corrected for multiples correlations.

## Data Availability

The datasets generated and analyzed during the current study are available in OSF repository and can be retrieved from osf.io/nwc6k.
