# Supplementary material for: Sound localization in noisy contexts: performance, metacognitive evaluations and head movements
Source: Cogn Res Princ Implic. 2024 Jan 8;9:4. doi: 10.1186/s41235-023-00530-w (PMC10774233; doi:10.1186/s41235-023-00530-w)
Supplement: Supplementary file 1 — Additional file 1. Supplementary Table 1. Spearman correlations between head movements variables (Number of reversals, Space explored with the head and Approaching behaviour) collected during the sound localization phase and the metacognitive variables of estimated effort and self-efficacy collected in the exposure phase. Values are not corrected for multiples correlations. [file 41235_2023_530_MOESM1_ESM.docx]

**Supplementary Table 1**

| NATURE | Estimated Effort | Estimated Self-efficacy | Number of reversals | Space explored with the head | Approaching behaviour |
| --- | --- | --- | --- | --- | --- |
| Estimated Effort |  |  |  |  |  |
| Estimated Self-efficacy | -0.57*** |  |  |  |  |
| Number of reversals | 0.15 | -0.25 |  |  |  |
| Space explored with the head | 0.22 | -0.20 | 0.72*** |  |  |
| Approaching behaviour | -0.10 | 0.45 | -034 | 0.03 |  |

| TRAFFIC | Estimated Effort | Estimated Self-efficacy | Number of reversals | Space explored with the head | Approaching behaviour |
| --- | --- | --- | --- | --- | --- |
| Estimated Effort |  |  |  |  |  |
| Estimated Self-efficacy | -0.70*** |  |  |  |  |
| Number of reversals | -0.43* | 0.13 |  |  |  |
| Space explored with the head | -0.31 | 0.11 | 0.76*** |  |  |
| Approaching behaviour | -0.22 | 0.18 | -0.30 | -0.10 |  |

| COCKTAIL PARTY | Estimated Effort | Estimated Self-efficacy | Number of reversals | Space explored with the head | Approaching behaviour |
| --- | --- | --- | --- | --- | --- |
| Estimated Effort |  |  |  |  |  |
| Estimated Self-efficacy | -0.58*** |  |  |  |  |
| Number of reversals | -0.8 | 0.04 |  |  |  |
| Space explored with the head | 0.05 | 0.12 | 0.67*** |  |  |
| Approaching behaviour | 0.05 | 0.10 | 0.-0.46 | -0.9 |  |

Spearman correlations between head movements variables (Number of reversals, Space explored with the head and Approaching behaviour) collected during the sound localization phase and the metacognitive variables of estimated effort and self-efficacy collected in the exposure phase. Values are not corrected for multiples correlations.
